# Fundamental Study of Phased Array Ultrasonic Cavitation Abrasive Flow Polishing Titanium Alloy Tubes

**DOI:** 10.3390/ma17215185

**Published:** 2024-10-24

**Authors:** Yuhan Dai, Sisi Li, Ming Feng, Baiyi Chen, Jiaping Qiao

**Affiliations:** 1 College of Mechanical and Electrical Engineering, Wenzhou University, Wenzhou 325035, China; 2 Rui’an Graduate College, Wenzhou University, Wenzhou 325206, China; 3 Marine Engineering College, Jimei University, Xiamen 361021, China; 4 Shenzhen KiSONICS Technology Co., Ltd., Shenzhen 518107, China

**Keywords:** phase control, ultrasonic abrasive flow, titanium alloy tube, polishing

## Abstract

A new method of machining ultrasonic cavitation abrasive flow based on phase control technology was proposed for improving the machining efficiency of the inner wall of TC4 (Ti-6Al-4V) titanium alloy tubes. According to ultrasonic phase control theory and Hertzian contact theory, a model of ultrasonic abrasive material removal rate under phase control technology was established. Using COMSOL Multiphysics 6.1 software, the phase control deflection effect, acoustic field distribution, and the size of the phase control cavitation domain on the inner wall surface were examined at different transducer frequencies and transducer spacings. The results show that the inner wall polishing has the most excellent effect at a transducer frequency of 21 kHz and spacing of 100 mm. In addition, the phased deflection limit was explored under the optimal parameters, and predictive analyses were performed for voltage control under uniform inner wall polishing. Finally, the effect of processing time on polishing was experimented with, and the results showed that the polishing efficiency was highest from 0 to 30 min and stabilized after 60 min. In addition, the change in surface roughness and material removal of the workpiece were analyzed under the control of the voltage applied, and the experimental results corresponded to the voltage prediction analysis results of the simulation, which proved the viability of phase control abrasive flow polishing for the uniformity of material removal of the inner wall of the tube.

## 1. Introduction

Titanium alloy has many advantages, such as high strength, high toughness, good corrosion resistance, excellent biocompatibility, and low density. Thus, tubular titanium alloy structures were widely used in aerospace [1], biomedical [2], automotive [3], oil and gas transportation [4], and marine desalination systems [5]. Due to the application fields, most of them are large-sized and complex-shaped tubes [6]. Meanwhile, because the manufacturing processes of titanium alloy tubes mainly rely on rolling, the difficulty in controlling rolling temperature can easily lead to surface sub-damage defects [7], which have huge impacts on the mechanical properties and service life of titanium alloys [8]. Aerospace, biomedical, and other fields need to use precision fittings to transport high-purity fluid media; the surface quality of the inner wall of the fittings has high requirements, usually needing to ensure that the inner wall of the fittings is smooth and clean, with a surface roughness of 0.2 μm or less. Therefore, it is necessary to polish the inner surface of titanium alloy tubes to reduce surface roughness and eliminate surface defects [9]. However, the inner surface of titanium alloy tubes is difficult to polish due to lower thermal conductivity and elastic modulus [10]. Traditional mechanical polishing methods cannot satisfy the current polishing efficiency and accuracy requirements, and they are labor-intensive. Additionally, the tubular shape increases the difficulties of polishing [11,12]. Therefore, it is necessary to use new methods to address the above challenges.

In recent years, electrochemical polishing, laser polishing, magnetorheological polishing, abrasive flow polishing, and ultrasonic cavitation abrasive flow polishing have attracted researchers’ attention [13,14,15,16]. Electrochemical polishing is a technique that passes current through an electrolyte solution or electrolyte membrane onto the surface of a workpiece to improve surface quality. Liu J et al. [17] introduced a micro-abrasive high-speed flow-assisted electrochemical polishing (ECFAP) method, and experiments showed that the average surface roughness of the ECFAP-treated TC4 (Ti6Al4V) specimens treated for 10 min at an optimal voltage of 3 V was 0.0953 μm. Although electrochemical polishing on the surface of titanium alloy is efficient polishing, there is the effect of welding slag in the tube, attached residues, and serious pollution of the environment. Laser polishing shows potential for selective polishing of metals and alloys and is also used for finishing titanium alloys, where surface morphology is altered by remelting, effectively improving surface quality without changing or affecting properties [18]. Although laser polishing is promising, remelting the surface may further exacerbate thermal residual stress and even lead to adverse changes in surface chemical properties [19]. Electrochemical polishing efficiently polishes the surface of titanium alloys while simultaneously removing weld slag and adhered residues inside the tubes. However, the metallic ions in the polishing solution severely pollute the environment [20].

To avoid crystal phase damage on polished surfaces, free abrasive polishing is commonly used for tube polishing. Abrasive flow polishing is the most common polishing technique used in factories. Abushanab et al. [21] studied TC4 (Ti6Al4V) titanium alloy using the abrasive flow polishing technique and showed that water pressure in the range of 2000~3000 bar decreased the roughness with increasing water pressure due to sufficient removal of unwanted material and increased jet energy. All the differently oriented surfaces were polished down to a uniform roughness of Ra1-3 μm [22]. Magnetorheological polishing applies low pressure to the workpiece surface, avoiding subsurface damage. It is often used for precision polishing of irregularly shaped workpieces [23]. However, the polishing consistency and stability of magnetorheological fluids are affected by polishing time.

Ultrasonic abrasive flow polishing is a technology that uses cavitation energy to drive abrasives for surface polishing. Cavitation is the formation, growth, and collapse of cavitation bubbles [24]. High-speed micro-jets will generate to drive material removal when cavitation collapse of the free abrasive particles occurs [25]. Beaucamp et al. [26] developed a novel ultrasonic cavitation-assisted fluid jet polishing system. Ultrasonic cavitation directly generates microbubbles upstream of the nozzle outlet, resulting in a 380% increase in material removal rate (MRR). Tan and Yeo [27,28] used ultrasonic abrasive flow polishing to finish the internal surface of additively manufactured tubes, and the surface roughness was improved by 20%. Kumar et al. [29] investigated the micro-deburring mechanism of difficult-to-machine materials using ultrasonic cavitation-accelerated alumina abrasives. The results show that burrs of soft materials are reduced by 92% in 10 s. Du et al. [30] employed a spherical array focusing ultrasonic abrasive for single-point removal polishing of smooth quartz glass. The study indicates that the increase in abrasive concentration increased the impact density and material removal rate, while excessive abrasive concentration increased the impeding effect between abrasive particles and reduced the material removal rate. However, the efficiency of ultrasonic cavitation polishing of titanium alloy is not satisfactory.

To improve the polishing efficiency of precision titanium alloy tubes, it is necessary to modify the polishing mechanism so that the impact of abrasives shifts more towards micro-cutting and plowing actions. The most effective method is the ultrasonic phased array. The ultrasonic phased array consists of multiple ultrasonic tools arranged in a certain pattern and sequence, and the delay time of the excitation signal sent to each array element is controlled electronically [31]. With ultrasonic phased array, the cavitation zone will be moving on the tube surface, leading to continuously plowing and micro-cutting the workpiece surface. This research employs ultrasonic phased array to enhance the efficiency of ultrasonic cavitation abrasive flow polishing of precision titanium alloy tube, hereafter named ultrasonic phased abrasive flow polishing (UPAFP).

## 2. Experimental Setup and Experimental Model

### 2.1. Polishing Principle

**Figure 1:**
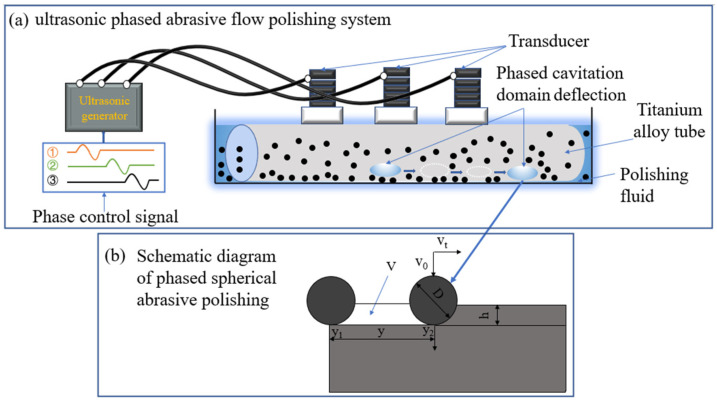
Principle of UPAFP.

The principle of UPAFP is shown in Figure 1. The ultrasonic generator controlled the ultrasonic transducer through phase difference. During the polishing process, the polishing fluid formed cavitation bubbles under the action of the acoustic field, which was generated by phase control. The collapse of the cavitation bubbles produced shock waves and drove abrasive to impact the inner wall at high speed. Abrasive generates material removal on the surface of the workpieces through scratch mode. Under the influence of the phased ultrasonic generator, the gathered cavitation domain could be controllably moved. The erosion of material removed would create more plowing and micro-cutting. Due to the overall height symmetry of the experimental setup, only the law of the phase control cavitation domain moving from the middle of the inner wall to the right is discussed to facilitate simulation and experimental analysis.

### 2.2. Experimental Setup

**Figure 2:**
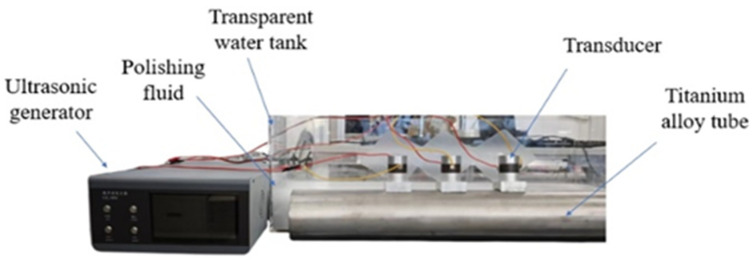
Experimental system of UPAFP.

To complete the smooth constant polishing pressure machining on tube inner surfaces, the UPAFP system was built, as shown in Figure 2. The ultrasonic cavitation abrasive flow polishing with ultrasonic phased array system mainly consists of four parts: piezoelectric ceramic ultrasonic transducer, ultrasonic generator, water tank, and titanium alloy tube.

The titanium alloy tube of TC4 (Ti6Al4V) was selected (outer diameter: 120 mm, wall thickness: 3 mm, and length: 600 mm). Before connecting to the tube, acoustic coupling gel (epoxy resin) was coated on the contact surface of the transducer to ensure that no air bubbles were retained between the transducer and the tube to maintain rigid contact. The transducer was placed in the tank together with the tube after the connection was completed. The polishing fluid was made from aluminum abrasive grains configured with deionized water at a concentration of 5 wt%. The polishing solution submerged the whole tube during the polishing process. The polishing solution should keep the solute particles in suspension during the experimental polishing process to achieve better polishing results. The control of the cavitation domain is achieved by a custom-made ultrasonic generator that excites the ultrasonic transducer with time delay signals of different phase differences. Under the excitation of the ultrasonic generator, the acoustic waves generated by the transducer will form a controlled pooled acoustic field in the inner wall region of the tube.

### 2.3. Polishing Pressure Model

The total radiation acoustic field of *N* array elements could be obtained by the following Equation [32]:

(1) $$p=if\rho \sum_{n=1}^{N}\iint_{S}u{\left(x\right)}_{n}\frac{e^{-jkr_{n}}}{r_{n}}ds_{n}$$

where *p* is the acoustic pressure of the synthetic acoustic field at the phase array, *ρ* is the density of the acoustic field medium, k = 2*π*f/*c* is the angular wave number, *S* is the whole region of the ultrasonic vibration surface, *ds* is the integration unit on the ultrasonic vibrating face, *r* is the distance from the point in the acoustic field to the integration unit, and *u*(*x*) is the vibration function on the ultrasonic vibrating face.

Cavitation can occur with a cavitation threshold, and the cavitation threshold is about 50 kPa under room temperature [33]. After cavitation, the impact velocity *v*_0_ of the abrasive particles is as follows:

(2) $$v_{0}=\frac{pe^{-\alpha h}}{\rho c}$$

where *c* is the speed of sound in the acoustic field medium, *α* is the attenuation coefficient and *h* is the ultrasonic propagation distance.

The experimental material used was aluminum abrasive; hence, spherical abrasive was studied. Abrasive could use tangential velocity *vt* to grind and micro-cut workpieces, improving polishing efficiency. Under the action of ultrasonic vibration, an abrasive with a diameter *D* and a density *ρ*_0_ impacted the workpiece surface at a normal velocity *v*_0_ and normal force *F*. According to the Hertz contact theory [34], the mathematical expression for the polishing depth *h* of abrasive impact could be obtained as follows:

(3) $$h={\left(\frac{9F^{2}}{8DE^{2}}\right)}^{\frac{1}{3}}$$

where *E* is the equivalent modulus of elasticity with the expression:

(4) $$\frac{1}{E}=\frac{1-\mu_{1}^{2}}{E_{1}}+\frac{1-\mu_{2}^{2}}{E_{2}}$$

where *μ*_1_ is the Poisson’s ratio of the abrasive and *μ*_2_ is the Poisson’s ratio of the workpiece. *E*_1_ is the modulus of elasticity of the abrasive and *E*_2_ is the modulus of elasticity of the workpiece.

UPAFP belongs to non-contact ultrasonic polishing [35]. The mass of a single abrasive is *m* = (*πD*^3^*ρ*_0_)/6, where the diameter is *D*, and the mass density is *ρ*_0_. The relationship between polishing depth and normal velocity was derived from the law of kinetic energy.

(5) $$h=\frac{1}{2}{\left(\frac{5\pi }{4}\rho_{0}E\right)}^{-\frac{2}{5}}v_{0}^{\frac{2}{5}}D$$

The cross-sectional area of contact between a single abrasive and the workpiece was *S*_0_ = 4*πDh*, resulting in the removal volume of a single abrasive in time *dt* is obtained as follows:

(6) $$V=S_{0}\cdot dy$$

*dy* is the contact path of abrasive per time *dt*.

(7) $$dy=v_{t}dt$$

Due to the approximate constancy of contact stress between two objects during the process of material plastic deformation [36], the removal rate of the phase array polishing material could be obtained as:

(8) $$MRR=\frac{V}{t}=\frac{\rho_{0}\pi D^{3}v_{0}^{2}}{24\sigma_{s}t}\int_{y_{1}}^{y_{2}}v_{t}dy$$

**Figure 3:**
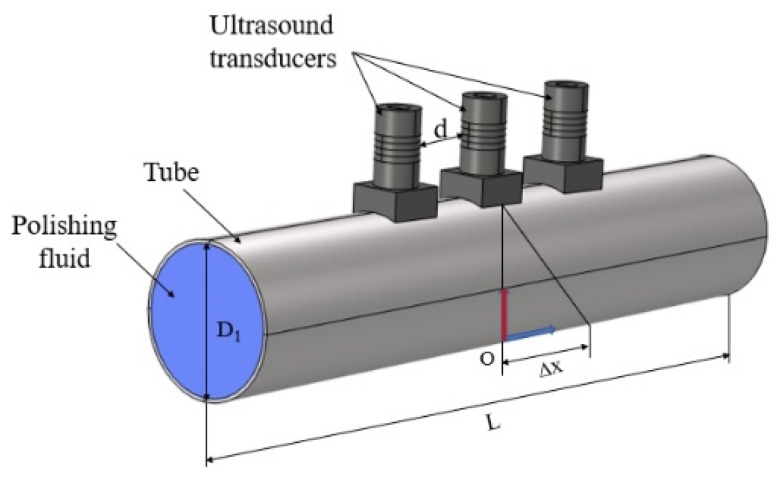
3D model of UPAFP.

Three ultrasonic transducers were selected to simulate the movement of the polishing area with COMSOL Multiphysics 6.1. TC4 (Ti6Al4V) titanium alloy tube was chosen as the workpiece. The 3D model is shown in Figure 3, where d is the spacing between transducers, and ∆x is the deflection distance from coordinate origin O for the test point. The ultrasonic phase array tube model consists mainly of ultrasonic transducers placed on the outer surface of the titanium tube. The transducers and tubes used were matched to the dimensions and material properties of the experimental samples. The model ignored the contact between the epoxy resin and the cover plate. The coupling of the transducer contact surface with the tube surface was achieved by applying acoustic coupling gel between the transducer and tube contact surface. The specific physical fields used are ‘Pressure Acoustic Frequency Domain’, ‘Solid Mechanics’, ‘Electrostatics’, ‘Piezoelectric Effect’, and ‘Acoustic-structure boundary’.

For pressure acoustics, this is assigned to the fluid domain and uses the ultrasonic pressure wave equation:

(9) $$\frac{1}{\rho c^{2}}\frac{\partial^{2}p_{t}}{\partial t^{2}}+\nabla \cdot \left(-\frac{1}{\rho }\right)\left(\nabla p_{t}-q_{d}\right)=Q_{m}$$

where *ρ* is the total density, *p_t_* is the total pressure, *ρc*2 is the bulk modulus, *q_d_* is the dipole source and *Q_m_* is the monopole source.

The solid mechanics physical field is applied to the rest of the model because these components are solids. The physical field is controlled by the Navier equations:

(10) $$\rho \frac{\partial^{2}u}{\partial t^{2}}=\nabla \cdot FS+F_{v}$$

where *ρ* is the fluid density, *u* is the velocity of the fluid, *F* is the deformation gradient, *Fv* is a body force, and *S* is the second Piola-Kirchhoff stress tensor.

Electrostatic phenomena are only included in piezoelectric ceramic rings where the phase control signal is applied using the following equation:

(11) $$\nabla \cdot D=\rho_{v}$$

(12) E=−∇V

where ∇*·D* is the electric charge density, *ρv* is the electric charge concentration and *E* is the electric field due to the electric potential *V*.

Multiphysics field modules are assigned to couple pressure acoustic and solid mechanics physical fields at the acoustic-structural boundary between the fluid and solid domains, as well as to couple solid and electrostatic physical fields into piezoelectric effects.

Subsequently, a free tetrahedral mesh was used to divide the entire solid domain and the water domain. To accurately calculate the acoustic pressure waves in the inner water domain, it should be ensured that there are at least five meshes within a wavelength, so the maximum size of the model mesh was set to one-fifth of the smallest wavelength generated by the ultrasonic transducer, which was 48 mm.

According to Equation (8), the main parameters affecting the polishing effect were phased array. Therefore, this work focused on discussing the control of tube phased acoustic pressure under different phase parameters. The control parameters were used to optimize the polishing effect on the inner wall of the tube. Based on the principle of ultrasound phased array [37], the main parameters affecting phased acoustic pressure deflection and focusing were transducer frequency and transducer spacing. The simulation parameters are shown in Table 1.

## 3. Simulation Results and Discussion

### 3.1. Effect of Transducer Frequency on Acoustic Field

**Figure 4:**
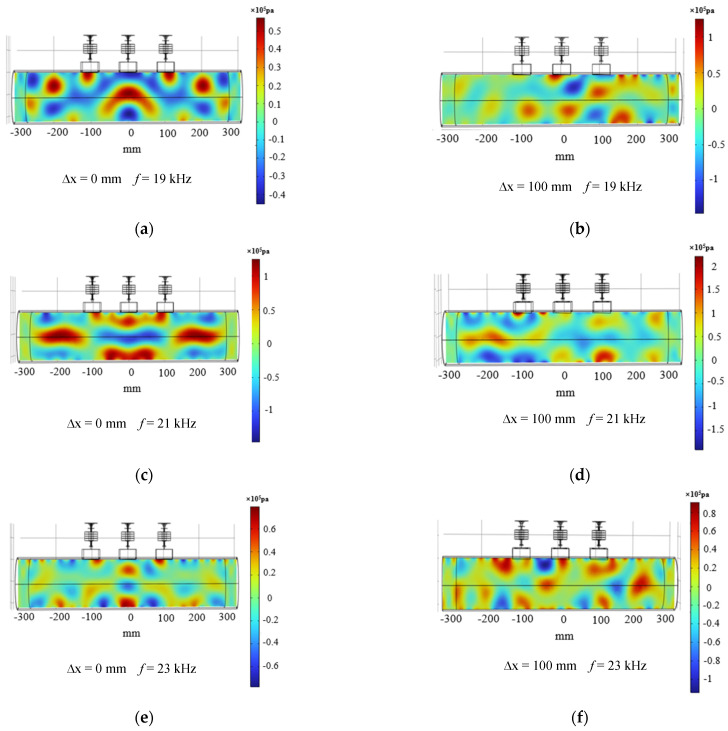
Distribution of acoustic pressure in the inner wall of the phased duct at different frequencies. (**a**) Frequency 19 kHz, phase control deflection distance ∆x = 0 mm, (**b**) Frequency 19kHz, phase control deflection distance ∆x = 100 mm, (**c**) Frequency 21 kHz, phase control deflection distance ∆x = 0 mm, (**d**) Frequency 21 kHz, phase control deflection distance ∆x = 100 mm, (**e**) Frequency 23 kHz, phase control deflection distance ∆x = 0 mm, (**f**) Frequency 23 kHz, phase control deflection distance ∆x = 100 mm.

Since the trends of acoustic field changes caused by increasing frequency were similar, the acoustic pressure distribution diagrams with *f* = 19–23 kHz were selected here for comparative analysis. As shown in Figure 4, where the frequency of Figure 4a,b was 19 kHz, the frequency of Figure 4c,d was 21 kHz, and the frequency of Figure 4e,f was 23 kHz, the simulation results of the phase deflection distance ∆x = 0, 100 mm.

**Figure 5:**
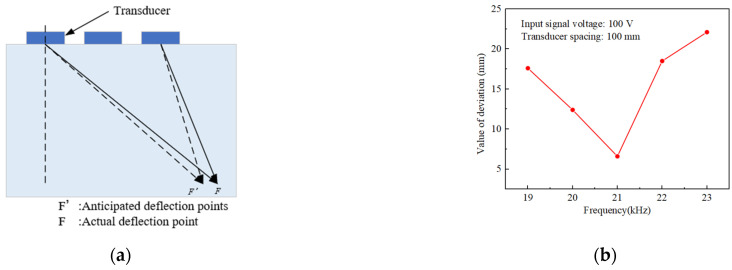
(**a**) Principle of phase control deviation (**b**) Deflection distance at ∆x = 100 mm value of deviation with frequency.

It could be observed from simulation Figure 4a–c that a good phase control effect was gradually revealed as the frequency increased, and the pooled acoustic pressure appeared at ∆x = 0 mm. At the same time, the size of the phase control region decreases with increasing frequency. When the phase control region was shifted to the right to ∆x = 100 mm, it could be seen from Figure 4b,d,f that there was a certain deflection of the acoustic pressure there. This was because the smaller the deflection angle, the better the directivity of the ultrasound beam and the higher the acoustic pressure energy. As the deflection angle increases, the phase control point moves away from the source and decreases the directivity [38]. Therefore, to explore the change of phase control deviation with frequency when ∆x = 100 mm, Figure 5a principle of phase control deviation and Figure 5b change rule are obtained. From Figure 5b, it could be seen that only the value of deviation shows the change rule of decreasing and then increasing, and it has a more accurate phase control at 21 kHz, and the value of deviation is only 6.2 mm. Obviously, only the ideal state of phase array polishing to reach the precise effect, resulting in this deviation is often the acoustic pressure energy through the different media attenuation of the impact. Therefore, it is more in line with the expectation of the phase array effect to minimize the effect at 21 kHz.

**Figure 6:**
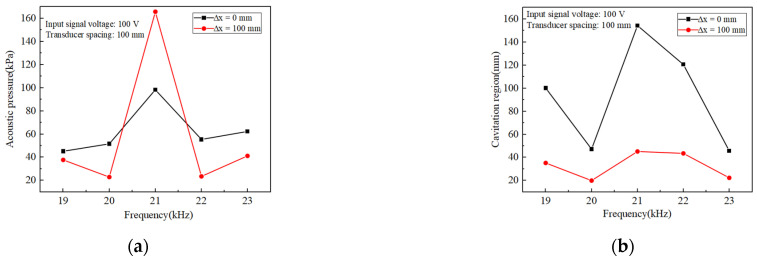
(**a**) Variation of acoustic pressure amplitude with frequency for different deflection distances (**b**) Size of phase control cavitation region with frequency for different deflection distances.

For comparison, statistics on the magnitude of the inner-wall phased acoustic pressure amplitude and the size of the cavitation domain range at five frequencies were carried out. Figure 6 shows the trend of acoustic pressure amplitude and range with frequency at two deflection distances for five different frequencies. From Figure 6a, it could be seen that at a phase array deflection distance of 0 mm, the amplitude of the acoustic pressure increased and then decreased as the frequency increased, and the amplitude of the acoustic pressure had a high gain at a frequency of 21 kHz. When the frequency was increased from 19 kHz to 21 kHz, the acoustic pressure increased by 164.2%, which was obviously changed. This was mainly because the resonant frequency of the designed transducer was near 21 kHz, where the transducer conversion efficiency is significantly increased compared to other frequency states, so there will be a larger acoustic pressure gain at 21 kHz. Therefore, in the actual machining process, it is necessary to ensure that the frequency is under the resonance frequency of the transducer to improve the effect of workpiece material removal [39]. When the phase array deflection distance was 100 mm, the same maximum acoustic pressure amplitude was found at 21 kHz with 339.5% gain compared to the lowest amplitude at other frequencies. Figure 6b shows that only at 21 kHz was the polishing area the largest, and at two different deflection distances, the area sums up to 196.3 mm. Therefore, in the actual transducer design, it is necessary to track its resonant frequency; at the transducer resonant frequency, transducer amplitude is large, and the vibration with the distance of the decay is slow. This facilitates the expansion of the cavitation domain and maximizes the acoustic pressure gain.

### 3.2. Effect of Transducer Spacing on Acoustic Field

The size of the transducer spacing is an important parameter affecting the phase control, and the change in its spacing size directly affects the effect of phase control. Too little spacing affects the limiting distance of the phase control deflection, and too much spacing may affect the amount of acoustic pressure at the deflection point. The changes in the transducer acoustic field and the cavitation domain range were discussed under the spacing of d = 80 mm, 90 mm, 100 mm, 110 mm, and 120 mm.

**Figure 7:**
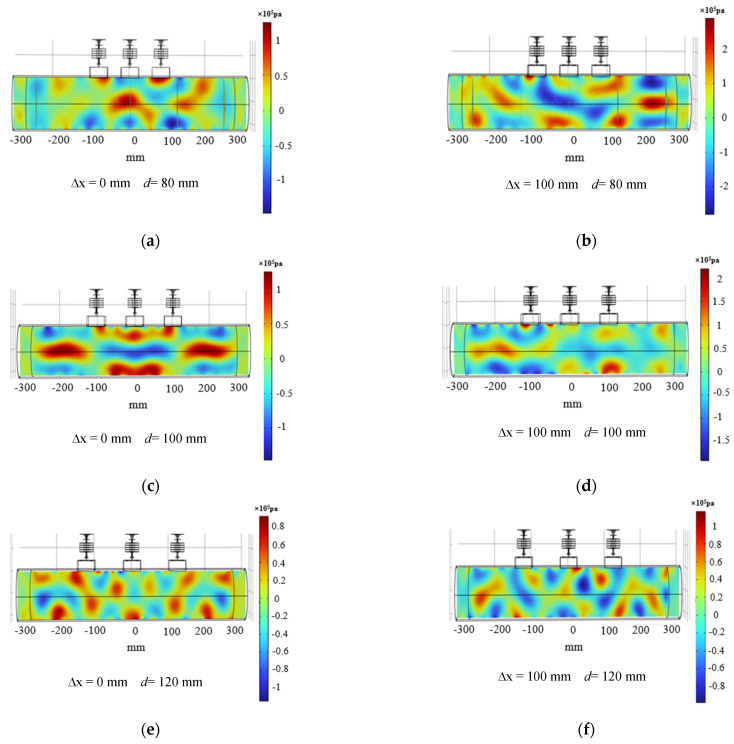
Distribution of acoustic pressure in the inner wall of a phased tube with different spacing. (**a**) Spacing 80 mm, phase control deflection distance ∆x = 0 mm, (**b**) Spacing 80 mm, phase control deflection distance ∆x = 100 mm, (**c**) Spacing 100 mm, phase control deflection distance ∆x = 0 mm, (**d**) Spacing 100 mm, phase control deflection distance ∆x = 100 mm, (**e**) Spacing 120 mm, phase control deflection distance ∆x = 0 mm, (**f**) Spacing 120 mm, phase control deflection ∆x = 100 mm.

Similarly, the acoustic pressure distribution plots with spacing of d = 80, 100, and 120 mm were selected here for comparative analysis. As shown in Figure 7, where the spacing was 80 mm in Figure 7a,b, 100 mm in Figure 7c,d, and 120 mm in Figure 7e,f, under conditions of ∆x = 0, 100 mm.

**Figure 8:**
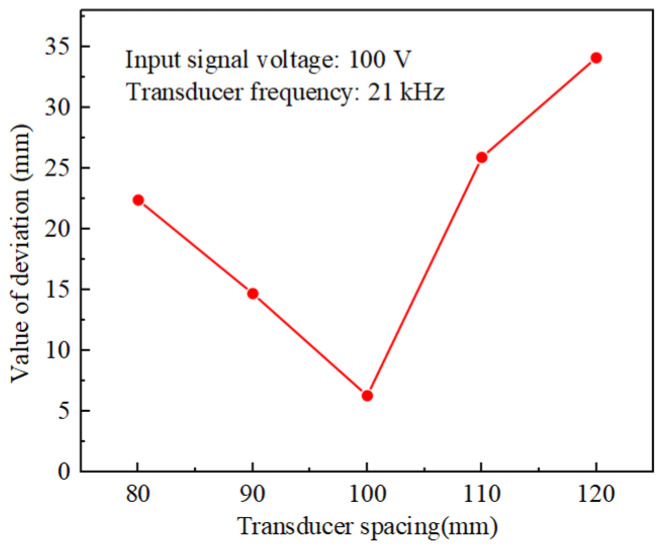
Deflection distance at ∆x = 100 mm value of deviation with transducer spacing.

An excellent gradual effect could be formed at the position where ∆x = 0 mm as the spacing of the transducer array increased in Figure 7a. When the control point moves to ∆x = 100 mm on the inner wall of the tube, the phase array deflection point fails to achieve good pooled acoustic pressure and has a certain deviation at the spacing of 80 mm, 100 mm, and 120 mm in Figure 7b,d,e. The reason for this phenomenon was due to the increase in deflection angle, which leads to a decrease in the directionality of the acoustic beam, as described in Section 3.1. Similarly, it could be seen that the deflection value is still the smallest at ∆x = 100 mm, compared with the phase array deflection values at a deviation distance ∆x = 100 mm under different spacing in Figure 8.

**Figure 9:**
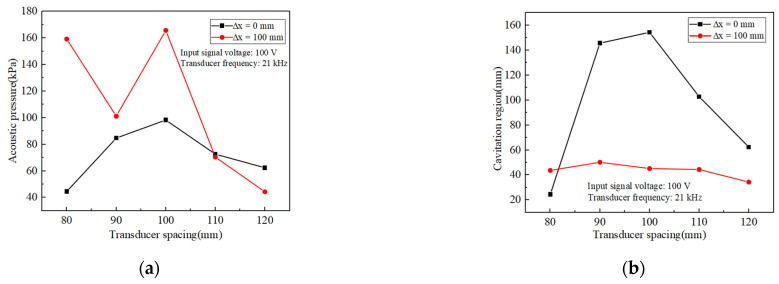
(**a**) Variation of acoustic pressure amplitude with transducer spacing for different deflection distances (**b**) Size of phase control cavitation region with transducer spacing for different deflection distances.

The trend of acoustic pressure amplitude and range with transducer spacing at two deflection distances for five sets of different transducer spacings was shown in Figure 9. When ∆x = 100 mm, the acoustic pressure amplitude at different phase array deflection distances was the largest. When ∆x < 100 mm and ∆x > 100 mm, acoustic pressure decreased significantly, and the acoustic pressure amplitude was lower than 50 kPa at ∆x = 80 mm and 120 mm. When ∆x = 0 mm, with the increasing spacing, the acoustic pressure amplitude shows the trend of first increasing and then decreasing, and at the spacing of 100 mm, the acoustic pressure amplitude shows the trend of first increasing and then decreasing. At 0 mm phase deflection, the acoustic pressure amplitude increased and decreased with the increasing pitch. The acoustic pressure amplitude was the highest at 100 mm pitch, which was 120.9% higher than the lowest acoustic pressure amplitude at most other pitches. When ∆x = 100 mm, the same transducer spacing of 100 mm had the largest acoustic pressure amplitude, compared with the lowest acoustic pressure amplitude of other spacing, with 274.5% gain. As shown in Figure 9b, the polished area was the largest at 100 mm spacing, and there was a huge decrease in the polished area below 100 mm and above 100 mm. The array spacing value must consider the tube length appropriately to find the optimal value.

### 3.3. Phase Control Deflection Limit and Tube Wall Acoustic Field Uniformity Control

**Figure 10:**
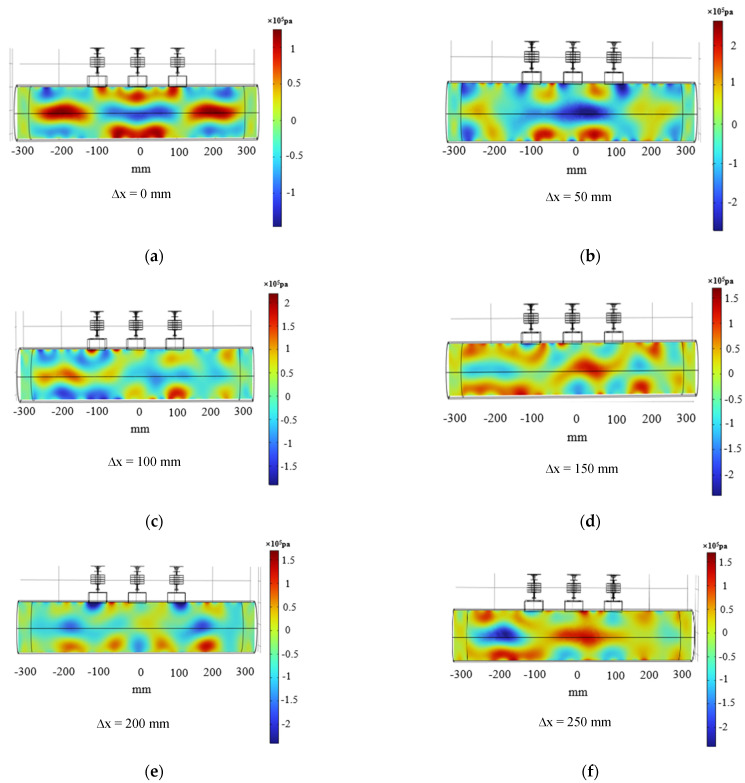
Distribution of acoustic pressure at different deflection distances in the inner wall of the tube with optimum parameters. (**a**) phase control deflection distance ∆x = 0 mm, (**b**) phase control deflection distance ∆x = 50 mm, (**c**) phase control deflection distance ∆x = 100 mm, (**d**) phase control deflection distance ∆x = 150 mm, (**e**) phase control deflection distance ∆x = 200 mm, (**f**) phase control deflection distance ∆x = 250 mm.

To explore the deflection effect, its rightward deflection distance ∆x = 0, 50, 100, 150, 200, 250 mm was simulated, and its simulation results were shown in Figure 10.

**Figure 11:**
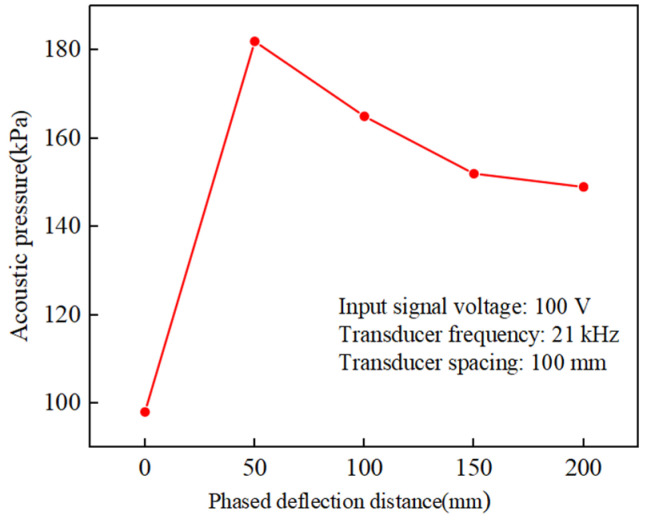
Variation curve of material removal rate with phase control deflection distance.

The acoustic pressure distribution in the inner wall of the tube at different deflection distances was shown in Figure 10. As could be seen from the Figure 10, a limiting range occurs as the deflection distance increased, with a deflection distance of ∆x = 200 mm being the limiting distance. Beyond 200 mm, the phase control effect cannot be achieved because the gradually increasing deflection angle was beyond the effective control range of the ultrasonic phase control. The surface acoustic pressure amplitudes at the boundary ∆x = 0, 50, 100, 150, and 200 mm in the simulated machining area were extracted and analyzed, and Figure 11 shows the variation curves of acoustic pressure amplitudes at different deflection distances. From the Figure 11, it could be seen that the acoustic pressure amplitude increases nonlinearly with the increase of deflection distance, and with the increase of the phase control deflection distance, the size of the acoustic pressure amplitude tends to increase from increasing to decreasing, and the size of the amplitude area range appears to decrease gradually to a flat trend. This behavior can be attributed to the fact that as the deflection point moves further away from the transducer resulting in more attenuation of the acoustic energy, the larger the amplitude region the smaller the overall energy.

In addition, this variation in the amplitude of the acoustic pressure during the phase control movement leads to an uneven phenomenon presented by the removal of the inner wall material, which affects the smoothness of the overall inner wall polishing of the tube. In the process of UPAFP, the phase control acoustic pressure can be controlled by controlling the amplitude of the output of the ultrasonic transducer. The amplitude of the output of the ultrasonic transducer increases with the increase of the output power of the ultrasonic power supply, and the actual power of the ultrasonic transducer increases with an increase in the input voltage. Therefore, the amplitude of the output of the ultrasonic transducer can be controlled by controlling the magnitude of the input signal voltage of the ultrasonic transducer, which in turn controls the magnitude of the phase control acoustic pressure at different deflection distances. By controlling the voltage, the amplitude of the acoustic pressure at different deflection points was made uniform at 98.3 kPa to achieve a uniform material removal rate at different deflection distances. To further investigate the relationship between voltage and acoustic pressure, simulation software was used to debug the acoustic pressure to uniformity, resulting in the voltage inputs at different phase control distances as shown in Table 2.

From Table 2, it can be observed that the control voltage with the deflection distance trend shows a pattern of decreasing and then increasing, and the control voltage input was the smallest at a deflection distance of about 50 mm, which was opposite to the simulation results of the acoustic pressure change trend. This trend can be attributed to the gradual increase in the phase control energy focusing at deflection distances <50 mm; however, at deflection distances >50 mm, the increasing impact of acoustic attenuation requires a gradual increase in the control voltage input.

## 4. Experimental Results and Discussion

**Figure 12:**
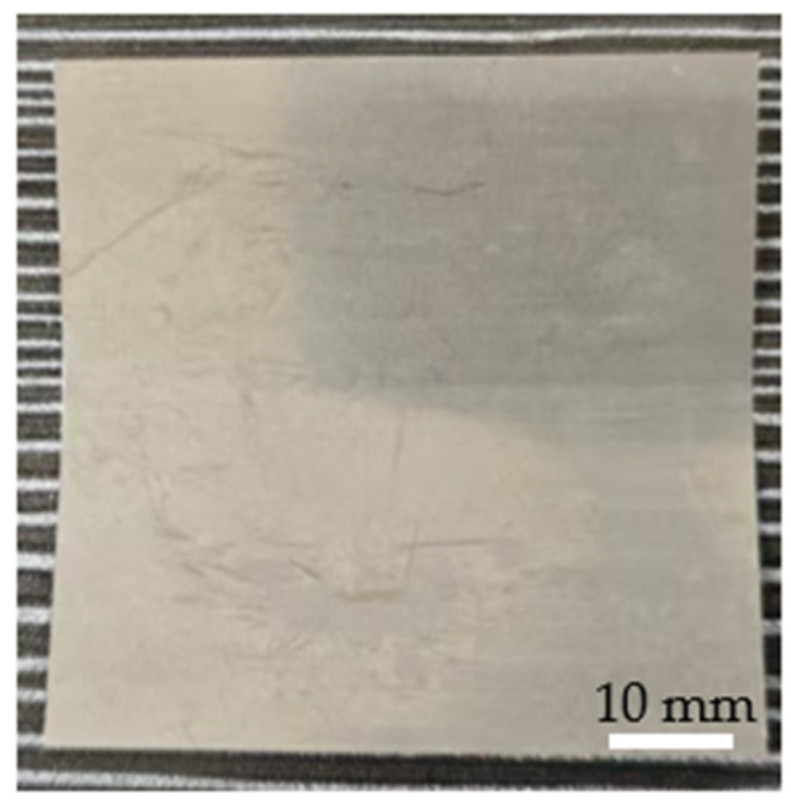
TC4 (Ti6Al4V) workpiece.

The effect of the change in material removal mechanism under the action of ultrasonic phase control on the removal characteristics of titanium alloy tube materials was experimentally investigated. Considering the high hardness of titanium alloy tube material, aluminum oxide abrasive was chosen to prepare the polishing solution. To facilitate the subsequent observation processing and analysis of the experimental samples, the experiments were carried out by bending the TC4 (Ti6Al4V) alloy sheet in the form of the sample adhering to the inner wall as shown in Figure 12. The workpieces were custom-sized research-grade TC4 titanium alloy sheets. A 3D surface profiler (WM-100-S) was used to measure the polished surface three times and calculate the average value. The specific condition parameters are shown in Table 3.

### 4.1. Effect of Changes in Processing Time

**Figure 13:**
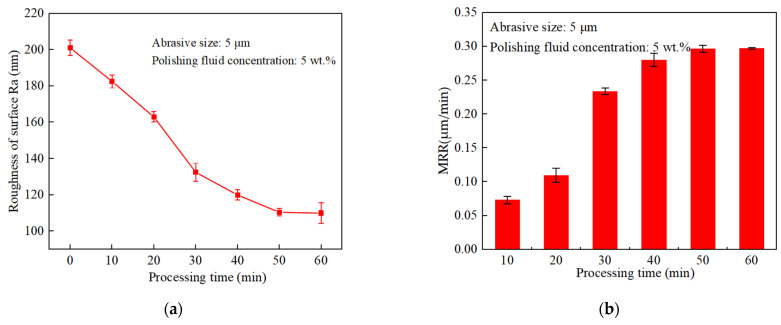
Effect of machining time on surface roughness and material removal rate of workpiece; (**a**) Effect of machining time on roughness; (**b**) Effect of machining time on material removal.

The abrasive slurry was a mixture of alumina, abrasive, and deionized water. The particle size of the alumina abrasive was 5 μm and the mass concentration was 5%. The ultrasonic system was used to polish the TC4 (Ti6Al4V) alloy samples from 0 to 60 min, and the material surface roughness and material removal rate (MRR) varied with the processing time as shown in Figure 13. Based on the experimental results, it can be seen that the surface roughness of the workpiece decreases the fastest when the processing time was from 0 min to 30 min, and the decrease in roughness from 30 min to 60 min slows down and tends to be stable. Similar to the roughness, the material removal rate has the largest rate of change from 0 to 30 min, and after 30 min, it also decreases gradually with an increase in machining time and tends to be stable. In the end, the overall roughness was improved to less than 0.2 μm by the surface requirement of the precision tube, and the roughness was reduced by 46.5% after 60 min of polishing.

**Figure 14:**
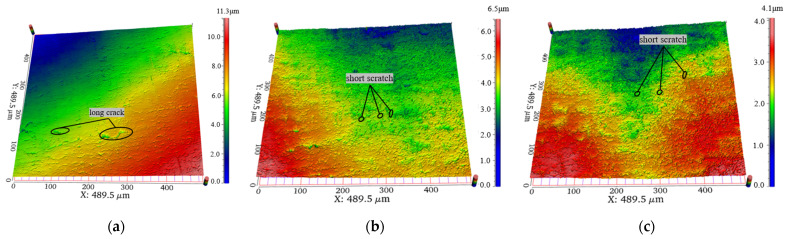
Comparison of morphology at different processing times; (**a**) 0 min; (**b**) 30 min; (**c**) 60 min.

A 3D surface profiler (WM-100-S) was used to observe the TC4 (Ti6Al4V) alloy samples. The 3D surface morphology of TC4 (Ti6Al4V) alloy samples before and after abrasive impact under phase control ultrasonic cavitation was obtained as shown in Figure 14. Figure 14 shows several surface morphologies at processing times of 0, 30, and 60 min, from which it can be seen that some irregular long cracks of 20~80 μm, which were part of the original morphological features on the surface of the workpieces, were removed in large quantities after 30 min of processing, and were replaced by a great number of new fine short scratches of less than 5 μm.

This short scratch machining with a large number of scratches and more material removal volume proved that plastic removal of the workpiece surface was achieved under the action of phase control ultrasound and that the removal effect was due to the impact of the abrasive particles on the workpiece surface under the vibratory excitation of phase control ultrasound. However, with an increase in the processing time, the polishing effect was close to the limit, and further extension of the polishing time cannot significantly improve the material removal rate. From the comparison of the morphology at processing times of 30 min and 60 min, it can be seen that the increase in such small short scratches in the latter 30 min increment was not significant; the morphology tends to stabilize, and there will be no significant improvement in the material removal efficiency, which further results in the surface roughness tending to be stabilized.

### 4.2. Effect of Changes in Voltage Control

**Figure 15:**
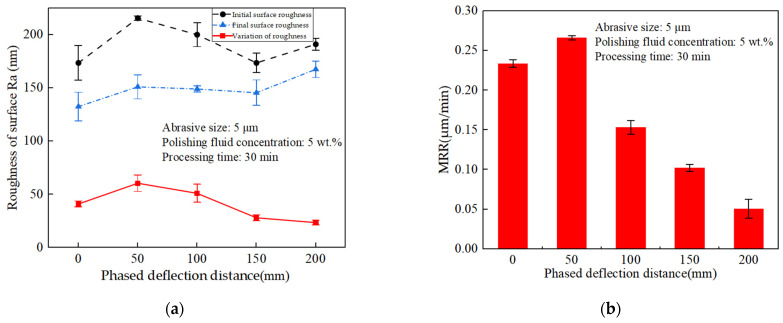
Effect of constant voltage on roughness variation and material removal rate at different phase control deflection distances (**a**) Effect of machining time on roughness (**b**) Effect of machining time on material removal.

According to the simulation results in Figure 11, it can be seen that when the voltage input value was fixed, the phase control deflection process to the right, and the size of the acoustic pressure amplitude presented a trend from increasing to decreasing. To verify this result, the processing was carried out at different phase control deflection distances of 0, 50, 100, 150, and 200 mm with constant voltage input, and the change rule of roughness difference before and after polishing at different phase control deflection distances as well as the effect of material removal rate (MRR) were observed; the results are shown in Figure 15. From Figure 15a, it can be seen that the amount of roughness change shows a pattern of first increasing and then decreasing; at deflection distances <50 mm, it gradually increases and reaches the peak, and at >50 mm, it gradually decreases. This rule of change is also consistent with the simulation results. Similarly, in Figure 15b, the MRR change rule also shows an increasing to decreasing trend, and the deflection distance of 50 mm had the largest material removal rate, which was also consistent with the deflection distance at which the phased acoustic pressure energy was the largest and the strongest cavitation effect of the simulation results.

**Figure 16:**
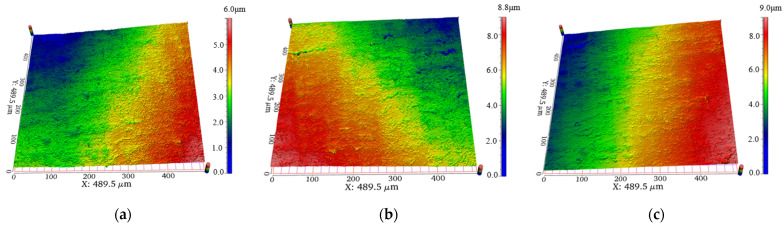
Comparison of morphology at different phase control deflection distances at constant voltage; (**a**) ∆x = 50 mm; (**b**) ∆x = 100 mm (**c**) ∆x = 150 mm.

Figure 16 shows the surface morphology after machining at several deflection distances for a constant voltage input. The machined morphology at the three different deflection distances shows a similar morphology to that of Figure 14, with a large number of short and shallow scratches as the main morphological feature. However, comparing the three kinds of morphology, it can be seen that the number of shallow and short scratches in Figure 16a was slightly higher than that in Figure 16b and higher than that in Figure 16c, and the morphology change rule at this point also confirms the change rule of the roughness difference and MRR at different phase control deflection distances.

**Figure 17:**
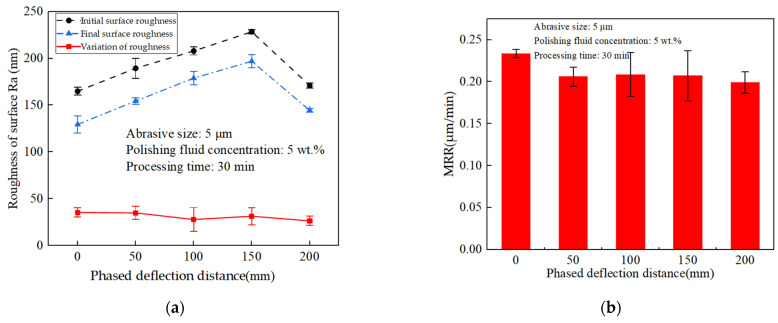
Effect of voltage control on roughness variation and material removal rate at different phase control deflection distances; (**a**) Effect of machining time on roughness; (**b**) Effect of machining time on material removal.

However, this effect for the actual tube polishing could not achieve a uniform smooth effect; different phase control distances require the application of voltage control to maintain uniform polishing. By the voltage input law in Table 2, the different phase control deflection distances of 0, 50, 100, 150, and 200 mm were processed, to observe the different phase control deflection distance polishing before and after the change rule of the roughness difference and the influence of the MRR; the results are shown in Figure 17. From Figure 17a, it can be seen that the roughness variation amount remains approximately the same at each phased deflection distance under the control of voltage input. In addition, the MRR variation rates in Figure 17b were all around 0.2 μm/min, which indicates that this voltage control could achieve the overall uniform polishing of the inner wall of the phased channel and confirms the simulation results.

**Figure 18:**
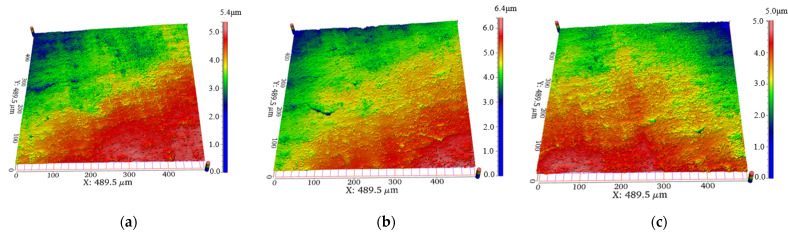
Comparison of morphology at different phase control deflection distances at the voltage control; (**a**) ∆x = 50 mm; (**b**) ∆x = 100 mm; (**c**) ∆x = 150 mm.

Figure 18 shows the surface morphology after processing at several deflection distances under controlled voltage. Comparing the three morphologies, it could be seen that the morphologies of the three at different phase control deflection distances were similar, and the overall surface was filled with a large number of short and shallow scratches left by the phase control polishing, which perfect illustrated its ability to achieve uniform polishing of the tube under the voltage control.

From the above experiments, it can be concluded that the surface of precision titanium alloy tubes can be effectively improved by the UPAFP method, and a typical removal feature on the surface of the workpiece was observed. Long cracks were removed from the surface of the original workpiece, while a large number of shallow short scratches were left behind. The synergistic effect of cavitation and micro-abrasive particles plays a crucial role in this, with the collapse of bubbles generating shock waves and micro-jets. Both shock waves and micro-jets cause the micro-abrasive particles in the liquid medium to acquire a certain velocity, and this velocity has a micro-cutting effect on the surface of the machined workpiece [40]. This type of machining trace, due to its large number, shows a greater ability to remove material. The main reason for this is the significant change in the material removal machining mechanism with the addition of phase control. This phase control movement is similar to that of grinding, where the controlled movement of the abrasive particles results in visible scratches on the surface of the workpiece. It can be attributed to the fact that the removal of traces is driven horizontally by the phase control movement of a large number of abrasive grains, which in turn creates a scouring, ploughing effect on the surface of the workpiece and enhances the removal of material from the surface of the workpiece.

## 5. Conclusions

In this study, UPAFP was carried out on the inner wall of a titanium alloy tube. The optimum parameters of the phase control were determined by finite element simulation. The phase control deflection limit was derived at the optimum parameters. Finally, the removal mechanism of UPAFP was studied through experimental research. The surface roughness and microscopic local morphology of TC4 (Ti6Al4V) titanium alloy specimens were observed. The conclusions were as follows:

(1) A phase control ultrasonic abrasive flow polishing device based on phase control was designed to achieve the polishing of titanium alloy tubes. Based on the phase control acoustic field theory and Hertzian contact theory, a material removal model for phase control abrasive flow polishing was established;
(2) Simulations were carried out to investigate the acoustic pressure effect of a phased tube with different parameters. The results show that the inner wall of the tube was polished with the best acoustic pressure amplitude benefit and polishing range at a transducer of 21 kHz and a pitch of 100 mm;
(3) Under the parameters of the transducer (21 kHz frequency and 100 mm spacing), the trend of the phase control deflection limit and the variation of the acoustic pressure amplitude with the deflection distance were simulated. The results show that the acoustic pressure amplitude varies in the range of 0~200 mm phase control deflection limit, which increases and then decreases. To obtain the uniformity of inner wall polishing, the voltage control was analyzed by studying the magnitude of the input voltage to the control transducer;
(4) The surface roughness and material removal rate fluctuated with the change in processing time. With the increase of processing time, the surface roughness of the workpiece decreases gradually, the polishing efficiency is the highest in 0~30 min, and it was stable after 60 min. Through the voltage control experiment, the roughness change and material removal effect of inner wall polishing with phase control deflection distance of 0~200 mm were analyzed, and this regulation corresponds to the results of the simulation. The feasibility of phase control abrasive flow polishing for uniformity of material removal from the inner wall of the tube is verified.

## Figures and Tables

**Table 1:** Simulation parameters.

Simulation Parameters	Values
Material of hole inner wall	TC4 (Ti6Al4V)
Transducer frequency *f*/(kHz)	19~23
Transducer spacing *d*/(mm)	80~120
Signal voltage *U*/(V)	100
Acoustic velocity in liquid *c*/(m/s)	1500
Fluid density *ρ*/(kg·m^−3^)	3100

**Table 2:** Voltage control data for deflection distance change.

Deflection Distance/mm	Input Voltage/V
0	100
50	60
100	68
150	78
200	80

**Table 3:** Experimental conditions.

Parameter	Values
Input voltage (V)	100
Input frequency (kHz)	21
Transducer array spacing (mm)	100
Abrasive size (μm)	5
Polishing fluid concentration (wt.%)	5
Processing time (min)	0~60
Workpiece	TC4 (Ti6Al4V)
Size of workpiece (mm)	50 × 50 × 2

## Data Availability

Data are all contained within the article. All data are fully available without restriction.
